# Exposure of Tumor-Associated Macrophages to Apoptotic MCF-7 Cells Promotes Breast Cancer Growth and Metastasis

**DOI:** 10.3390/ijms160611966

**Published:** 2015-05-26

**Authors:** Na Zhou, Yizhuang Zhang, Xuehui Zhang, Zhen Lei, Ruobi Hu, Hui Li, Yiqing Mao, Xi Wang, David M. Irwin, Gang Niu, Huanran Tan

**Affiliations:** 1 Department of Pharmacology, Peking University, Health Science Center, Beijing 100191, China; E-Mails: zhouna@bjmu.edu.cn (N.Z.); onepiecezyz1989@gmail.com (Y.Z.); xuehuizhang@bjmu.edu.cn (X.Z.); huruobi@bjmu.edu.cn (R.H.); lihui@bjmu.edu.cn (H.L.); maoyiqing@bjmu.edu.cn (Y.M.); xixi1125@bjmu.edu.cn (X.W.); 2 Beijing N&N Genetech Company, Beijing 100082, China; E-Mail: tanlab232@gmail.com; 3 Department of Laboratory Medicine and Pathobiology, University of Toronto, Toronto, ON M5S 1A8, Canada

**Keywords:** cancer stem cells, apoptosis, TAMs, metastasis, IL-6, STAT3

## Abstract

Tumor-associated macrophages (TAMs) have been found to be associated with the progression and metastasis of breast cancer. To clarify the mechanisms underlying the crosstalk between TAMs and cancer stem cells (CSCs) in breast cancer recurrence and metastasis, we used a co-culture model of macrophages and apoptotic human breast cancer cell line MCF-7 cells to investigate the effects of TAMs on MCF-7 *in vitro* and *in vivo*. Macrophages co-cultured with apoptotic MCF-7 had increased tumor growth and metastatic ability in a nude mouse transplantation assay. The macrophages exposed to apoptotic cells also induce an increase in the proportion of CD44^+^/CD24^−^ cancer stem-like cells, as well as their proliferative ability accompanied with an increase in mucin1 (MUC1) expression. During this process, macrophages secreted increased amounts of interleukin 6 (IL-6) leading to increased phosphorylation of signal transducers and activators of transcription 3 (STAT3), which likely explains the increased transcription of STAT3 target genes such as TGF-β1 and HIF-1α. Our results indicate that when cancer cells endure chemotherapy induced apoptosis, macrophages in their microenvironment can then activate cancer stem cells to promote cancer growth and metastasis by secreting IL-6, which activates STAT3 phosphorylation to regulate the transcription of its downstream target genes.

## 1. Introduction

Breast cancer is one of the most threatening diseases in females worldwide, with high incidence and fatality rates, accounting for approximately 23% (1.38 million) of all new cancer cases annually [1]. Metastasis and recurrence are common occurrences for breast cancer patients, despite surgical ablation, chemotherapy and radiotherapy diminishing or even eradicating the visible primary focus [2,3]. Tumor metastasis consists of a series of discrete biological processes that move a tumor cell from a primary neoplasm to a distant location [4]. Cancer cells cannot finish all of these steps by themselves; metastatic breast cancer development depends upon essential contributions from the tumor microenvironment, which consists of adipocytes, fibroblasts, a wide range of hematopoietic cells, as well as newly formed blood and lymphatic vessels, and their associated cells [5]. Tumor associated macrophages (TAMs) are a predominant component of the tumor mass in malignant carcinoma, particularly in breast cancers, where they can represent up to 50% of the tumor mass, and appear to be pivotal for orchestrating breast cancer development [6].

Accumulating evidences implicate TAMs in promoting metastasis of tumor cells to distant sites [7,8]. Removal of macrophages from many tumor models slowed the rate of tumor progression to the most malignant stage and reduced the rate of metastasis [9,10]. Macrophages appear to influence cancer metastasis at many stages in the processes. TAMs secrete extracellular material (ECM)-degrading enzymes, such as cathepsins, matrix metalloproteinases (MMPs), and serine proteases, which can proteolytically degrade the intracellular matrix, and thus loosen the fibrous connective tissue surrounding the tumor, enabling tumor cells to escape from the tumor mass and allow local invasion or entry to the circulation to initiate dispersion to a distant site [11]. In addition, TAMs can induce angiogenesis, which will not only supply nutrients and oxygen but also increase the availability of routes of entry into the systemic bloodstream, as newly formed vessels are highly permeable and have incomplete basement membranes [12]. Macrophages also enhance the ability of tumor cells to enter blood vessels through colony-stimulating factor (CSF-1) and epidermal growth factor (EGF) receptor signaling [13]. Recently, *in vitro* research has demonstrated an alternative mechanism for the promotion of breast cancer cell invasion by TAMs might be through macrophage-secreted exosomes, which would deliver invasion-potentiating miRNAs to breast cancer cells [14].

Little research has been conducted concerning the role of macrophages in cancer recurrence and metastasis after chemotherapy. Chemotherapy often leads to apoptosis of cancer cells, which potentially modify the physiological state of TAMs. To further examine the role of TAMs in the initiation and progression of metastasis, here, we established a post-chemotherapy cancer microenvironment model using the breast cancer cell line MCF-7 cultured in medium from a co-culture of macrophages and apoptotic breast cancer cells. We show that macrophages play a crucial role in promoting breast cancer relapse and metastasis after chemotherapy through a secreted factor.

## 2. Results

### 2.1. Macrophages Could Increase the Proportion of CD44^+^/CD24^−^ Cancer Stem Cells after Co-Culture with Apoptotic MCF-7 Cells

**Figure 1:**
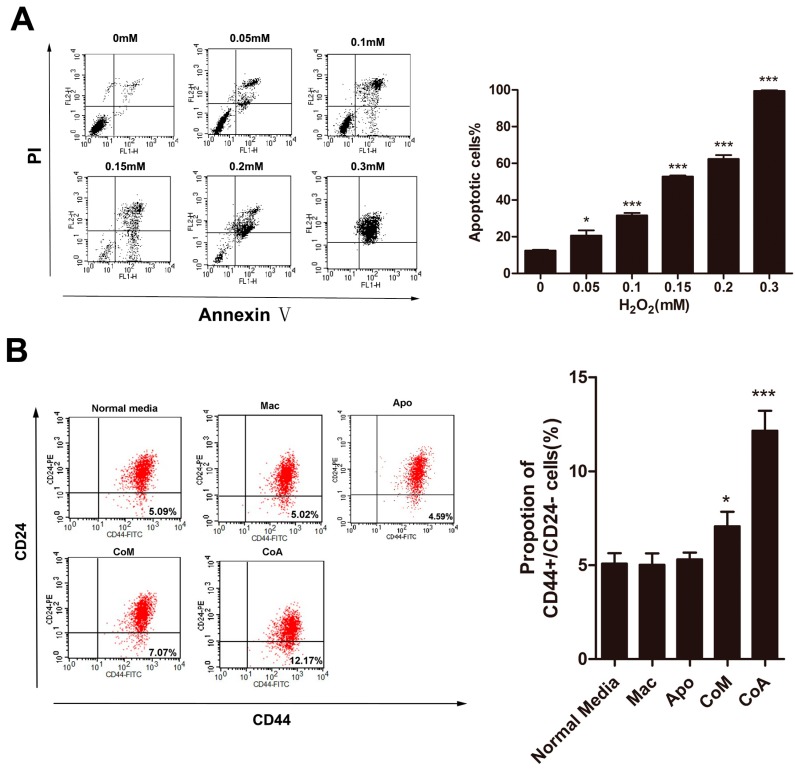
Analysis of CD44^+^/CD24^−^ subpopulations in MCF-7 cells. (**A**) Analysis of the apoptotic effect of H_2_O_2_ on MCF-7 cells. Percentage of apoptotic cells is the sum of the events in the upper right and lower right quadrants; (**B**) Flow cytometry analysis and quantification of the size of the CD44^+^/CD24^−^ subpopulation of MCF-7 cells treated with different types of conditioned media. Results are typical of three independent experiments. Data represent means ± S.E. (*n* = 3). * *p* < 0.05, *** *p* < 0.001 (Mac: conditioned media from macrophages alone; Apo: conditioned media from apoptotic MCF-7 cells alone; CoM: conditioned media from a co-culture of macrophages and MCF-7 cells; CoA: conditioned media from a co-culture of macrophages and apoptotic MCF-7 cells).

To mimic the breast cancer microenvironment treated with chemotherapy, we cultured MCF-7 with several different types of macrophage-conditioned media, media conditioned by only macrophages, by macrophages co-cultured with MCF-7 cells, and macrophages co-cultured with apoptotic MCF-7 cells. The co-culture with apoptotic cells is a model of the chemotherapy induced microenvironment. We used H_2_O_2_, which is toxic to cells and induces hypoxia [15], to induce apoptosis in MCF-7 cells. Levels of apoptosis induced by H_2_O_2_ were examined using flow cytometric analysis after PI-Annexin V co-staining of the cells. H_2_O_2_ induces an apoptotic effect on the human MCF-7 cell line, with 0.3 mM H_2_O_2_ generating nearly 100% apoptosis in these cells (Figure 1A). All further experiments used 0.3 mM H_2_O_2_ to generate apoptotic MCF-7 cells.

Cancer stem-like cells are a small subpopulation of cells found in all types of cancer, including breast cancer, which have been shown to be highly tumorigenic and exhibit some stem cell characteristics such as self-renewal and the ability to form tumor spheres [16]. CD44^+^/CD24^−^ cells are considered to be the cancer stem-like cells in breast cancer [17,18], and we use this group as an indicator of the proportion of cancer stem-like cells in our cell populations. In our research, 5.09% ± 0.55% of the MCF-7 cells grown in normal media are CD44^+^/CD24^−^. Growing MCF-7 cells in media conditioned by only macrophage cells (Mac group) or only apoptotic MCF-7 cells (Apo group) showed no significant difference in their percentage of CD44^+^/CD24^−^ MCF-7 (Figure 1B). When cells were grown in a conditioned media generated by normal MCF-7 cells and macrophages (CoM group) only a slight non-significant increase in the percentage of the CD44^+^/CD24^−^ was detected (Figure 1B). In contrast, when MCF-7 cells were cultured in conditioned media derived from the co-culture of macrophages and apoptotic MCF-7 (CoA group), our chemotherapy microenvironment model, a significant increase in the percentage of CD44^+^/CD24^−^ cells, up to 12.17% ± 1.05%, was seen (Figure 1B). These results imply that the conditioned media from the CoA group contains a secreted factor that is not present in the conditioned media from the Mac or CoM groups that increases the percentage of CD44^+^/CD24^−^ cells, potentially cancer stem-like cells, in a breast cancer cell line.

### 2.2. MCF-7 Cells Cultured in Conditioned Media from a Co-Culture of Macrophages and Apoptotic MCF-7 Show Increased Tumor Growth and Metastatic Ability in Vivo

**Figure 2:**
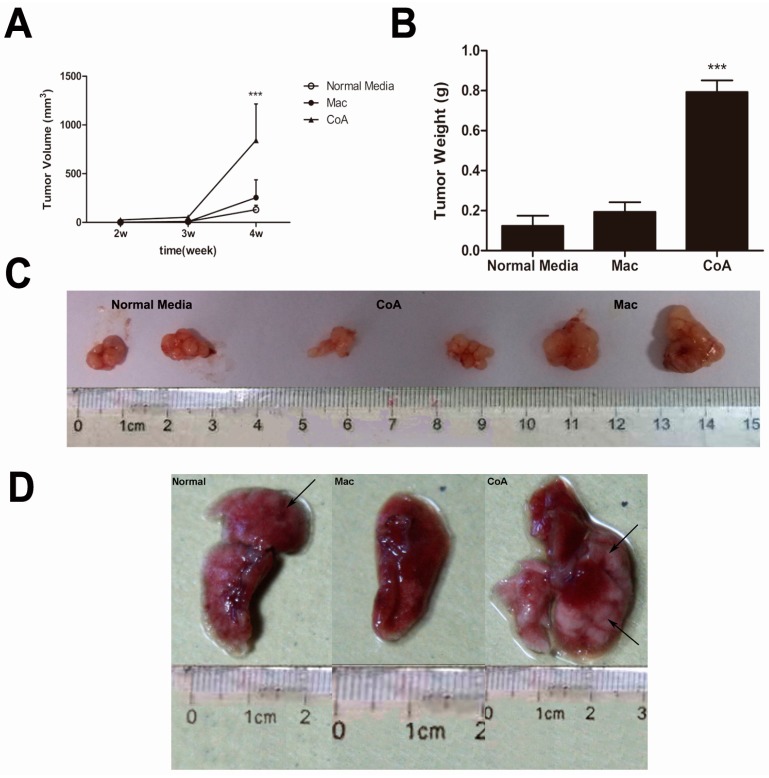

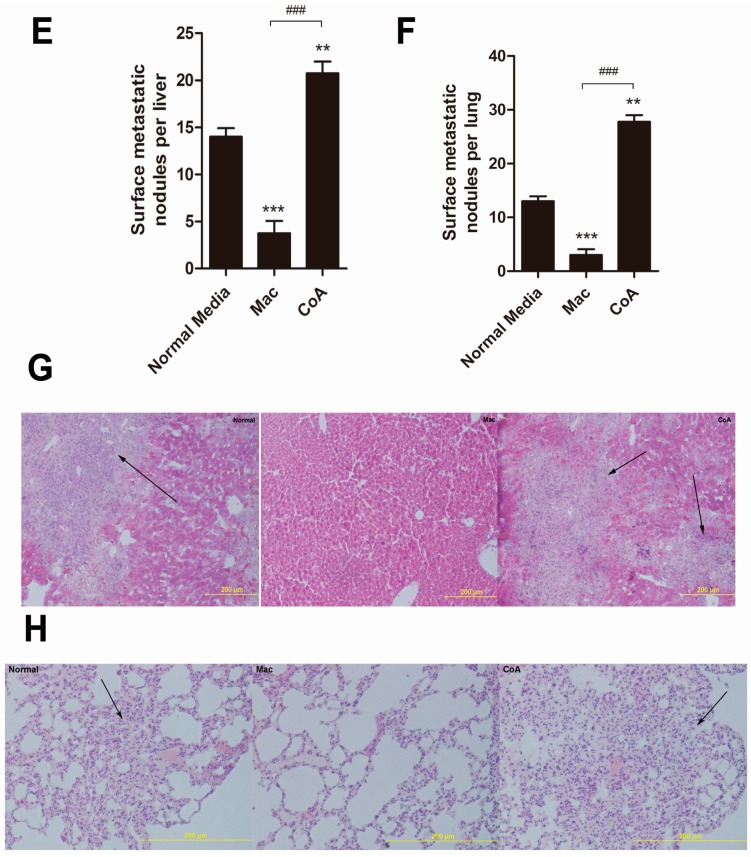
*In vivo* tumorigenicity and metastatic assay. (**A**) Growth curves for tumors generated by MCF-7 cells grown in three types of conditioned media. The width and diameter of each tumor were measured using calipers, and tumor volume was calculated using the formula ½ × a × b^2^, where “a” is the longer tumor axis and “b” is the shorter tumor axis; (**B**) Tumor weight was measured after excising from mice, *n* = 5; (**C**) Images of tumors from the three groups of mice; (**D**) Macroscopic view of nodules in the lungs from the three groups of mice; (**E**,**F**) Quantification of the metastatic nodules in the three groups of mice (*n* = 5); (**G**,**H**) Hematoxylin-eosin (HE) staining of paraffin sections from livers and the lungs of the three groups of mice. Metastases are indicated by the black arrows. ** *p* < 0.01, *** *p* < 0.001 *vs.* Normal media group; **^###^** *p* < 0.001 *vs.* Mac group.

To determine whether change in the proportion of CD44^+^/CD24^−^ cells induced by the co-culture of macrophages with apoptotic MCF-7 cells influence the malignancy of these cancer cells we tested the tumorigenicity of the MCF-7 cells exposed to the conditioned media using the nude mice model. MCF-7 cells cultured in normal media (*i.e.*, not conditioned) are capable of forming neoplasm by two weeks after subcutaneous injection, although they grow at a slow rate (Figure 2A). Macrophage conditioned media (Mac group) did not significantly change the growth rate of the neoplasm, however, MCF-7 cells exposed to conditioned media from macrophages grown with apoptotic MCF-7 cells (CoA group) grew at a significantly higher rate (Figure 2A). As expected, tumors from the CoA group were larger and heavier than those from the other two groups, with no difference in the tumor size or weight seen between the normal media and the Mac groups (Figure 2B,C). To further examine the metastatic properties of these cells we used the experimental metastatic model to examine the numbers of metastases formed in the liver and the lung. Visible and microscopic metastases were observed in both the normal media and the CoA groups but not in the Mac group (Figure 2D,G,H). When metastatic growths in the liver and the lung were examined, a very high number of foci were observed on the surface of liver and the lung in the CoA group, which was significantly higher than that seen in the Mac group (Figure 2E,F). These results suggest that the macrophage co-culture with apoptotic MCF-7 cells yields a conditioned media that not only promotes tumor growth *in vivo*, but also increases metastatic ability of the MCF-7 cells, whereas conditioned media from a macrophage culture alone inhibits tumor metastasis *in vivo*.

### 2.3. Macrophage Co-Culture with Apoptotic MCF-7 Cells Promotes the Proliferative Ability of CD44^+^/CD24^−^ Cancer Stem-Like Cells and Up-Regulates the Expression of Mucin 1

**Figure 3:**
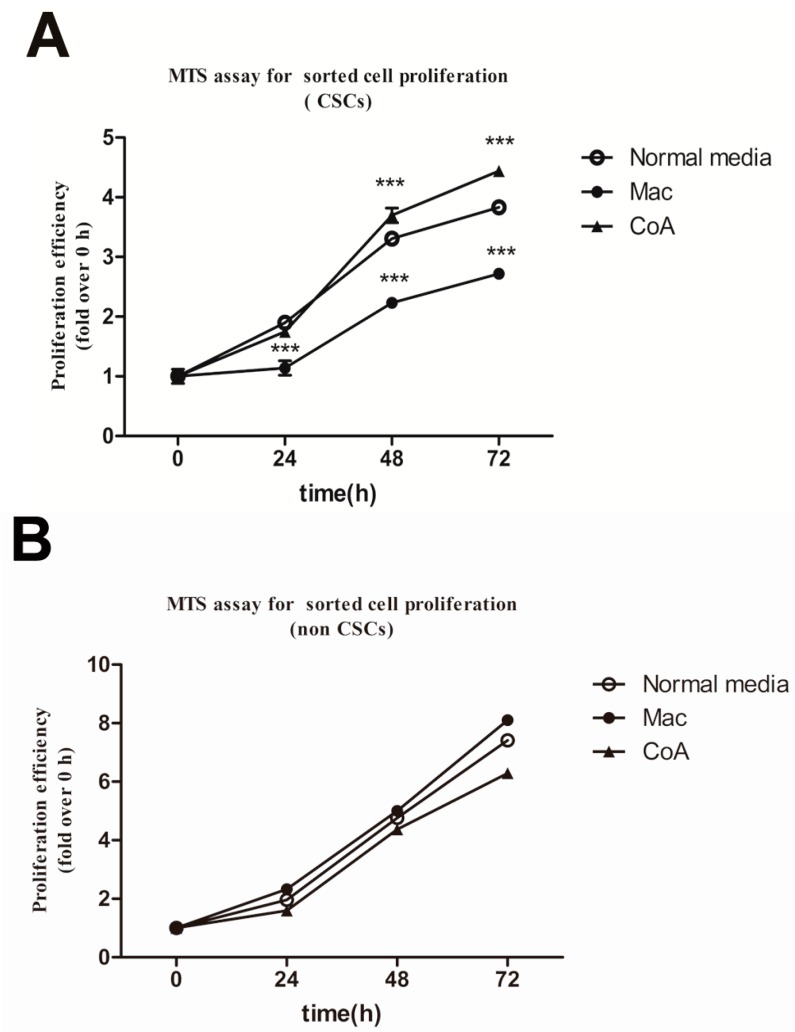

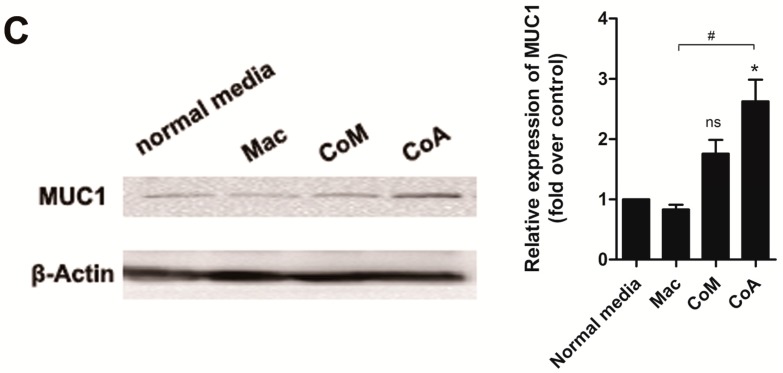
Analysis of the proliferative abilities of sorted CD44^+^/CD24^−^ and CD44^+^/CD24^+^ subpopulations of MCF-7 cells and Mucin1(MUC1) protein levels in MCF-7 cells cultured in four types of media. (**A**) 3-(4,5-dimethylthiazol-2-yl)-5-(3-carboxymethoxyphenyl)-2-(4-sulfophenyl)-2*H*-tetrazolium (MTS) assay examining the proliferative ability of the CD44^+^/CD24^−^ cells of the three groups. Results are typical of the three independent experiments. Data represent means ± S.E. (*n* = 3). *** *p* < 0.001 *vs.* the normal media group; (**B**) MTS assay examining the proliferative ability of the CD44^+^/CD24^+^ cells of the three groups. Results are typical of the three independent experiments. Data represent means ± S.E. (*n* = 3); (**C**) Western blot analysis of MUC1 levels. Band intensity was analyzed using Quantity One software and β-actin was used as a loading control. Protein levels were compared to the normal media group. Results are typical of three independent experiments. Data represent means ± S.E. (*n* = 3). * *p* < 0.05, *vs.* the Normal media group; ^#^ *p* < 0.05 *vs.* the Mac group; ns means no significance.

The tumorigenicity of cancer cells depends on the proportion and activity of cancer stem cells. The proportion of cancer stem cells in the tumor depends on the proliferative ability of both the cancer stem cells and the non-stem cells. CD44^+^/CD24^−^ cells have stem cell-like properties [19] and were used here as a marker of the cancer stem cell-like population. We sorted the CD44^+^/CD24^−^ cells and CD44^+^/CD24^+^ from MCF-7 cells that had been cultured in the different types of conditioned media and their proliferative ability was examined using the MTS assay. Growth of these two subpopulations was measured for 72 h. The CD44^+^/CD24^−^ cells from the CoA group displayed an increased proliferative rate, which was significantly more rapid at 48 and 72 h compared with the normal media group (Figure 3A). In contrast, the proliferative ability of the CD44^+^/CD24^−^ subpopulation from the Mac group was significantly decreased at all time points, including 24 h (Figure 3A). The proliferation ability of the CD44^+^/CD24^+^ subpopulation showed no significant difference among the three groups at any time point (Figure 3B). These results parallel the *in vivo* experimental metastasis assay, where the CoA group has increased metastasis and the Mac group decreased ability compared with the normal media cell group (Figure 2E,H).

In our previous research we found that mucin1 expression was increased during cancer stem cell activation induced by apoptosis [20]. Therefore, we examined whether mucin1 levels are associated with the augmentation of the proportion and proliferative ability of cancer stem-like cells from the CoA group. As shown in Figure 3C, MCF-7 cells from the CoA group have significantly higher levels of MUC1 protein compared to the normal media and Mac groups, with no significant difference observed among the other three groups.

### 2.4. Macrophage Interleukin 6 (IL-6)/Signal Transducers and Activators of Transcription 3 (STAT3) Signaling Pathway Is Activated after Co-Culture with Apoptotic Cancer Cells

**Figure 4:**
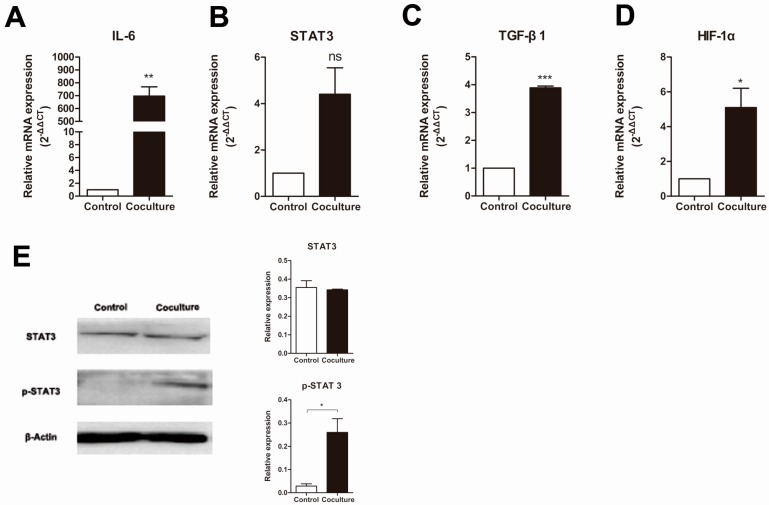
Changes in the expression of macrophage expressed genes in macrophages co-cultured with apoptotic MCF-7 cells. Relative amounts of mRNA of (**A**) IL-6; (**B**) STAT3; (**C**) TGF-β1; (**D**) HIF-1α mRNA was determined by real-time RT-PCR, where β-actin was used as an internal standard. Control is normal cultured macrophages while co-culture is macrophages grown with apoptotic MCF-7 cells. Results are typical of three independent experiments. Data represent means ± S.E. (*n* = 3). * *p* < 0.05, ** *p* < 0.01, *** *p* < 0.001, ns means no significance; (**E**) Analysis of the phosphorylation of STAT3 in macrophages (Control) or macrophages cocultured with apoptotic MCF-7 cells (Coculture) by Western blot. Bands were analyzed using Quantity One software with β-actin used as a loading control. Protein levels were compared to the normal group. Results are typical of three independent experiments. Data represent means ± S.E. (*n* = 3). * *p* < 0.05.

Since conditioned media generated by the co-culture of macrophages and apoptotic MCF-7 cells changes the proliferative ability of MCF-7 cells, this suggests that the macrophages secrete a protein into the conditioned media that induces proliferation. Macrophages, as immunocytes, secrete several kinds of cytokines, each of which plays different roles [6]. The cytokine interleukin 6 (IL-6) is produced by macrophages at sites of inflammation, especially acute inflammation, which occurs in cancer tumors during chemotherapy. Signal transducers and activators of transcription 3 (STAT3) is the main transcription factor through which IL-6 signals in target cells, where it regulates many genes including those that promote cancer proliferation and metastasis (e.g., TGF-β1) and angiogenesis (e.g., HIF-1α) [17]. To determine whether the IL-6 pathway had been activated in our chemotherapy microenvironment model we examined the levels of expression for the IL-6, STAT3, TGF-β1 and HIF-1α genes (Figure 4). As expected, the mRNA levels for IL-6, TGF-β1 and HIF-1α in the macrophage co-culture with apoptotic MCF-7 were much higher than in the control group (Figure 4A). However, no significant difference in the mRNA levels for STAT3 was observed between the two groups. However, since activation of the STAT3 signaling pathway is due to the level of phosphorylated STAT3, and not abundance of total STAT3, we then examined the phosphorylation status of STAT3 using a Western blot (Figure 4E). As expected from the real-time RT-PCR results (Figure 4B), no significant difference in the total protein levels of STAT3 was detected between the CoA and Mac groups, but activation of the STAT3 pathway, as assessed by the presence of a p-STAT3 band, was only detected in the macrophage co-culture with the apoptotic MCF-7 cell group, and not in the Mac group (Figure 4E). These results show that our chemotherapy microenvironment model yields macrophages that are activated and produce cytokines, such as IL-6, and thus are able to modify the proliferative and metastatic properties of cancer stem-like cells.

## 3. Discussion

A considerable number of patients with advanced stage carcinoma experience tumor recurrence or metastasis within a few years of treatment [21,22]. These disappointing facts are attributed to cancer stem cells [23]. Cancer cells, including cancer stem cells, live in complex microenvironments, where macrophages are the major component of the immune cells that infiltrate these tissues [13]. As guardians of the body, classically activated macrophages (M1) have the potential to contribute to the earliest stages of neoplasia [24], primarily due to the free radicals they produce that can lead to DNA damage. However, as tumorigenesis progresses, the tumor microenvironment markedly influences the tumor-associated macrophages, which change their physiology and promote tumor development. These macrophages are identified as M2 macrophages [25]. Tumor-associated macrophages (TAMs) have been implicated in tumor invasion, immune suppression and tumor metastasis [12,26]. In addition, TAMs have also been known to be a prognostic factor in many types of cancers and are involved in tumor recurrence [27,28]. Research on a spontaneous murine model of melanoma showed that these macrophages contribute to both post-surgical tumor relapse and to the growth of metastases, likely by stimulating a population of tumor-initiating cells, and that depletion of macrophages warrants exploration as an adjuvant to surgical resection [29]. Chemotherapy induces cancer cell apoptosis; our previous research indicated that chemotherapy-induced apoptosis may activate cancer stem-like cells (CD44^+^/CD24^−^) in MCF-7 and that upregulation of MUC1 occurred [20]. Thus we explored whether macrophages were involved in this progress. In our *in vitro* post-apoptosis microenvironment model, media from a co-culture of macrophages and apoptotic MCF-7 cells induced an increase in the abundance of CD44^+^/CD24^−^ cells, and also increased their proliferative ability (Figure 1B and Figure 3A), whereas co-culture of macrophages and MCF-7 just slightly increased the proportion of CD44^+^/CD24^−^ cells. In contrast, the proliferative ability of the CD44^+^/CD24^−^ subpopulation cultured in media conditioned by macrophages alone was inhibited (Figure 3A). Interestingly, an increase in MUC1 expression accompanies the increase in CD44^+^/CD24^−^ proportion (Figure 3B), whereas the same coordination was not observed in the CoM group. Although the relationship between MUC1 and cancer stem cells remains unexplored, a considerable number of recent studies have revealed that MUC1 is associated with cancer stem cells. MUC1 is expressed in human embryonic pluripotent stem cells and functions as a growth factor receptor to boost the proliferation of hESCs and prevents them from differentiating [30]. MUC1 is also involved in increasing the proportion of SP cells in the breast cancer cell line MCF-7 [31]. Our results also indicate that macrophages play an inherent role as an immunocyte to suppress cancer growth, but when co-cultured with cancer cells, they become the TAMs and stimulate enrichment of cancer stem cells. Importantly, after tumors have endured chemotherapy, which induces apoptosis, the macrophages produce a great change to the niche of the cancer stem cells, and stimulate cancer stem cells along with MUC1 expression.

MCF-7 cells from all three treatment groups formed tumors in our *in vivo* xenograft assays in nude mice, including those cultured in the macrophage conditioned media. However, tumors from the CoA group grew faster and became larger than those in the other two groups (Figure 2A). In our experimental metastasis model, MCF-7 cells from the CoA group metastasized to the liver and the lung more easily than the normal group, whereas no metastasis was observed for those from the Mac group (Figure 2D–H). These results suggest that MCF-7 cells from all the three groups contain cancer stem cells that can form neoplasms, but cancer stem cells from the CoA group were activated whereas cancer stem cells from the Mac group were inhibited by the macrophages.

The above results demonstrate that macrophages can promote cancer stem cell activation in a microenvironment that had endured cancer cell apoptosis. What happened to the macrophages when they were co-cultured with apoptotic cells and how do they regulate the activities of cancer stem cells? Considerable research has indicated that macrophages in a cancer microenvironment can promote cancer development by regulating the activities of both the cancer cells and the cancer stem cells [11,32]. However, research into the relationship between macrophages and cancer stem cells after cancer cell apoptosis is rare. It has been shown that TAMs serve as a source of key components in the inflammatory microenvironment, such as MFG-E8 and IL-6, in non-small-cell lung carcinoma (NSCLC) that trigger tumorigenicity and resistance to anticancer therapeutics by regulating CSCs activities [33]. Glioma cancer stem cells can induce p-STAT3 expression in macrophages, which in turn promotes cancer stem cells activation [34]. The IL-6 and STAT3 signaling pathway is one of the most important signaling pathways regulating physiological functions. In classical IL-6 signaling, the cytokine engages its receptor IL-6Ra at the cell surface, which is followed by the recruitment of the signal transducing receptor gp130 and activation of a Janus kinase (JAK1) that activates STAT3 by phosphorylation. Phosphorylated STAT3 dimerizes and then travels to the nucleus, there it initiates a transcriptional program [35]. Many target genes of STAT3 are involved in cancer, such as HIF-1α, which acts as an important factor in angiogenesis [36,37], and TGF-β1, which promotes cancer cell growth and metastasis [38]. In hepatocellular carcinoma, TAMs were shown to promote cancer stem cell-like properties with TGF-β1-induced epithelial–mesenchymal transition [39]. We examined the IL-6/STAT3 signaling pathway to determine whether it plays an important role in macrophage-induced activation of cancer stem cells after co-culture with apoptotic cancer cells. As expected, large amounts of IL-6 were secreted by macrophages co-cultured with apoptotic MCF-7 cancer cells, accompanied by the phosphorylation of STAT3 and the upregulation of the mRNA levels of TGF-β1 and HIF-1α (Figure 4). The IL-6/STAT3 signaling pathway also plays important roles in promoting the progression of hepatocellular carcinoma (HCC) by TAMs [40,41]. However, these studies were in patients with hepatocellular carcinoma who did not receive chemotherapy, thus the effects were only on present cancer growth and progression but not the circumstance after therapy. These results led us to conclude that macrophages stimulated by apoptotic MCF-7 cells secrete IL-6, which then activates STAT3 phosphorylation to regulate associated factors leading to the activation of the cancer stem cells and promotion of cancer development. Blockage of the IL-6/STAT3 signaling pathway might be a promising approach to decrease or prevent the recurrence and metastasis of cancer after chemotherapy.

## 4. Experimental Section

### 4.1. Mice

Female Institute of Cancer Research (ICR) mice and athymic nude mice (BALB/c nu-nu), 6–8 weeks of age, were purchased from the Laboratory Animal Science Department of Peking University Health Science Center. Mice were housed in a pathogen-free animal facility maintained at 25 °C and illuminated with a 12:12-h light-dark cycles. Mice were provided with standard rodent chow and water *ad libitum*. All animal protocols were approved by the Animal Care and Use Committee of Peking University (SYXK2011-0039, 12 December 2011–12 December 2016).

### 4.2. Mice Peritoneal Macrophages Isolation and Culture

Peritoneal macrophages were harvested from sacrificed ICR mice by flushing 5 mL of cold phosphate-buffered saline (PBS) into the peritoneal cavity. The fluid containing the macrophages was centrifuged and resuspended in Dulbecco’s modified Eagle’s medium (DMEM) (Life Technologies, New York, NY, USA) with 10% (*v*/*v*) fetal bovine serum (FBS) (Life Technologies). Cells were counted, plated in 6-well (5 × 10^5^ cells/well) flat-bottom culture plates (Costar, New York, NY, USA), and incubated for 2 h in a humidified atmosphere of 5% CO_2_ at 37 °C. After a 2 h incubation, to allow macrophages to adhere to the surface, macrophages were purified by discarding the supernatant and non-adherent cells. The remaining adherent cells were washed with warm PBS and incubated in DMEM containing 10% FBS before and during all experiments.

### 4.3. Generation of Apoptotic MCF-7

MCF-7 cells (ATCC^®^ HTB-22™) were cultured in DMEM high glucose (Life Technologies) supplemented with 100 U/mL penicillin, 100 μg/mL streptomycin, and 10% heat-inactivated FBS (Life Technologies), and maintained at 37 °C and 5% CO_2_. Cells (1 × 10^5^/mL) were plated into 7.5 cm plates containing 10 mL of cell culture medium. After 24 h of culture, MCF-7 cells were treated for 12 h with 0.3 mM H_2_O_2_ in full DMEM medium. Cell death was confirmed by flow cytometry, using Annexin V and propidium iodide (PI) co-staining (Biosea, Beijing, China).

### 4.4. Co-Culture Experiment and the Preparation of Conditioned Media

Purified peritoneal macrophages were cultured for 12 h, followed by the addition of MCF-7 cells or apoptotic MCF-7 cells at a ratio of 1:2 and co-culture was performed for 3 h at 37 °C. Cells were then pelleted by centrifugation and the supernatants from the co-culture as well as from a culture of macrophages alone were collected. The conditioned media, with normal media as control, was used to grow fresh MCF-7 cells for 24 h and then assessed for the next experiments.

### 4.5. Flow Cytometric Analysis and Fluorescence-Activated Cell Sorting (FACS)

Cells were collected and suspended in PBS, labeled with fluorescein isocyanate (FITC) mouse anti-human CD44 and phycoerythrin (PE) mouse anti-human CD24 (BD Pharmingen™, San Diego, CA, USA), with 5 μL of antibody per one million cells in a final volume of 300 μL. A total of about 1.0 × 10^6^ cells were incubated with these two antibodies for 0.5 h at 4 °C in the dark. Unbound antibody was washed off and the cells were analyzed on a BD fluorescence activated cell sorter (FACS) Calibur within 1 h of staining. Gating was established using the isotype control FITC-labeled mouse IgG2a and PE-labeled mouse IgG2b (BD Pharmingen™).

FACS was used to sort the differentiated cultured MCF-7 cells into two phenotypically distinct populations: CD44^+^/CD24^−^ and all other phenotypes. The labeling method and conditions were the same as those described above for the flow cytometric analysis.

### 4.6. Cell Proliferation Assay

Cell growth was examined with the MTS assay using Cell Titer 96^®^ Aqueous One Solution (Promega Biotech Co., Ltd., Madison, WI, USA) according to the manufacturer’s instructions. Briefly, cells were seeded at the density of 10,000 cells/well into 96-well plates. At the time of assay, 20 μL MTS was added to each well and incubated for 1–4 h. Absorbance was measured at 490 nm using a Vmax microplate reader (Bio-Rad, Hercules, CA, USA).

### 4.7. Nude Mice Xenografts Assay and Tumor Metastasis Assay

Cells (1 × 10^6^) suspended in 0.2 mL PBS were injected subcutaneously into the right armpit of nude mice. After 4 weeks, mice were sacrificed by cervical dislocation, photographs of the tumors were taken with a digital camera, and tumors were excised to determine their weight and volume.

The experimental metastasis model was performed as previously described with some modifications [42]. Briefly, BALB/c nu-nu mice were injected with a MCF-7 cell suspension containing 1 × 10^6^ cells via the tail vein. After 4 weeks, mice were sacrificed by cervical dislocation and the lungs and livers of the mice in each group were harvested, fixed in 4% paraformaldehyde and then dehydrated in 20% sucrose solution. Visible nodules were assessed under a dissection microscope. The fixed tissues were then embedded in paraffin, serially sectioned, and the HE-stained paraffin sections were examined to evaluate the presence and number of metastatic tumors.

### 4.8. Isolation of Total RNA and Quantitative RT-PCR

Total RNA was isolated using Trizol Reagent (Invitrogen, New York, NY, USA) according to the manufacturer’s instructions. cDNA was synthesized from 2 μg of total RNA using a high capacity cDNA reverse transcription kit (D6110A, TAKARA, Otsu, Japan). Amplifications were performed in the BIO-RAD Miniopticon™ Real-Time PCR Detection systemCFB-3120 using iQTM SYBR Green Supermix 170-8880 (Bio-Rad) with primers listed in Table 1. All annealing temperatures were listed in the Table 1. Transcription levels were normalized to those of β-actin.

**Table 1:** Primers used for real-time RT-PCR analysis of gene expression.

Gene	Primer	Tm (°C)
IL-6	F-CAACGATGATGCACTTGCAGA R-CTCCAGGTAGCTATGGTACTCCAGA	64
STAT3	F-TGCACCTGATCACCTTCGAGAC R-CCCAAGCATTTGGCATCTGAC	68
TGF-β1	F-TACGGCAGTGGCTGAACCAA R-CGGTTCATGTCATGGATGGTG	68
HIF-1α	F-GGACGATGAACATCAAGTCAGCA R-AGGAATGGGTTCACAAATCAGCA	68
β-actin	F-CATCCGTAAAGACCTCTATGCCAAC R-ATGGAGCCACCGATCCACA	60

### 4.9. Western Blot Analysis

Total protein was extracted in a buffer containing 50 mM Tris-HCl (pH 8.0), 150 mM NaCl, 0.02% NaN_3_, 1% SDS, 1 mM EDTA, 0.5% Sodium deoxycholate, 100 mg/mL PMSF, 1 mg/mL leupeptin and 1% NP-40. Protein concentration was measured using the BCA Protein Assay Kit (CW0014, CWBIO, Beijng, China), following the manufacturer’s instructions. For each sample, 60 mg of protein was separated by SDS-PAGE, at different concentrations due to the differences in molecular weight, at 80 V for 0.5 h or 120 V for 1 h using the Mini-PROTEAN 3 electrophoresis cell system (Bio-Rad). Proteins were then transferred to a PVDF membrane (Bio-Rad) by the semi-dry blotting method and the Dunn carbonate transfer buffer (consisting of NaCHO_3_ (10 mM), Na_2_CO_3_ (3 mM), and 20% methanol). Membranes were blocked for 2 h with 5% *w*/*v* nonfat dry milk and then incubated overnight at 4 °C with the primary antibodies, mouse anti-MUC1 (1:500; #4538CST, Boston, MA, USA), mouse anti-STAT3 (1:1000; CST, #9139), and mouse anti-phospho-STAT3 (1:1000; CST, #4113). Antibody binding was detected after incubation with HRP-linked secondary antibodies, with the membrane-bound antibodies visualized by luminal chemiluminescence ChemiDoc XRS (Bio-Rad) after reaction with super ECL plus (P1010, Applygene, Beijng, China).

### 4.10. Statistical Analysis

All experiments were conducted in at least 3 independent cultures. All data are expressed as means ± standard error of mean and analyzed with one-way ANOVA followed by the Tukey’s *post hoc* test for multiple comparisons. *p* values less than 0.05 were considered significant.
