# Zhou, N., *et al*. Exposure of Tumor-Associated Macrophages to ApoptoticMCF-7 Cells Promotes Breast Cancer Growth and Metastasis. *Int. J. Mol. Sci.* 2015, *16*, 11966–11982

**DOI:** 10.3390/ijms160922957

**Published:** 2015-09-22

**Authors:** Na Zhou, Yizhuang Zhang, Xuehui Zhang, Zhen Lei, Ruobi Hu, Hui Li, Yiqing Mao, Xi Wang, David M. Irwin, Gang Niu, Huanran Tan

**Affiliations:** 1 Department of Pharmacology, Peking University, Health Science Center, Beijing 100191, China; E-Mails: zhouna@bjmu.edu.cn (N.Z.); onepiecezyz1989@gmail.com (Y.Z.); xuehuizhang@bjmu.edu.cn (X.Z.); huruobi@bjmu.edu.cn (R.H.); lihui@bjmu.edu.cn (H.L.); maoyiqing@bjmu.edu.cn (Y.M.); xixi1125@bjmu.edu.cn (X.W.); 2 Beijing N&N Genetech Company, Beijing 100082, China; E-Mail: tanlab232@gmail.com; 3 Department of Laboratory Medicine and Pathobiology, University of Toronto, Toronto, ON M5S 1A8, Canada

The authors wish to change Figure 2 in Section 2 of their paper published in *IJMS* [1]. In Figure 2C, the tumor tissue of the Mac group was mixed up with that of the CoA group. The authors have carefully checked the original files and found that it was an inadvertent mistake in the published version of Figure 2. Figure 2 is revised as follows. The authors apologize for any inconvenience.

**Figure 2:**
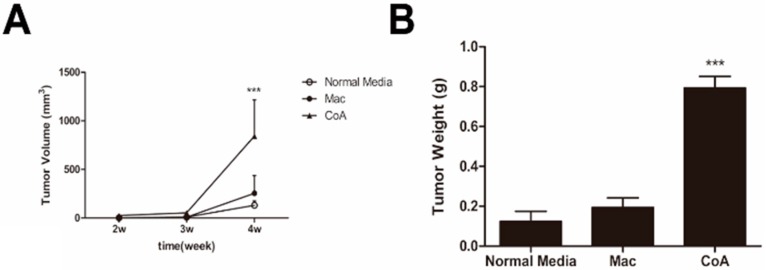

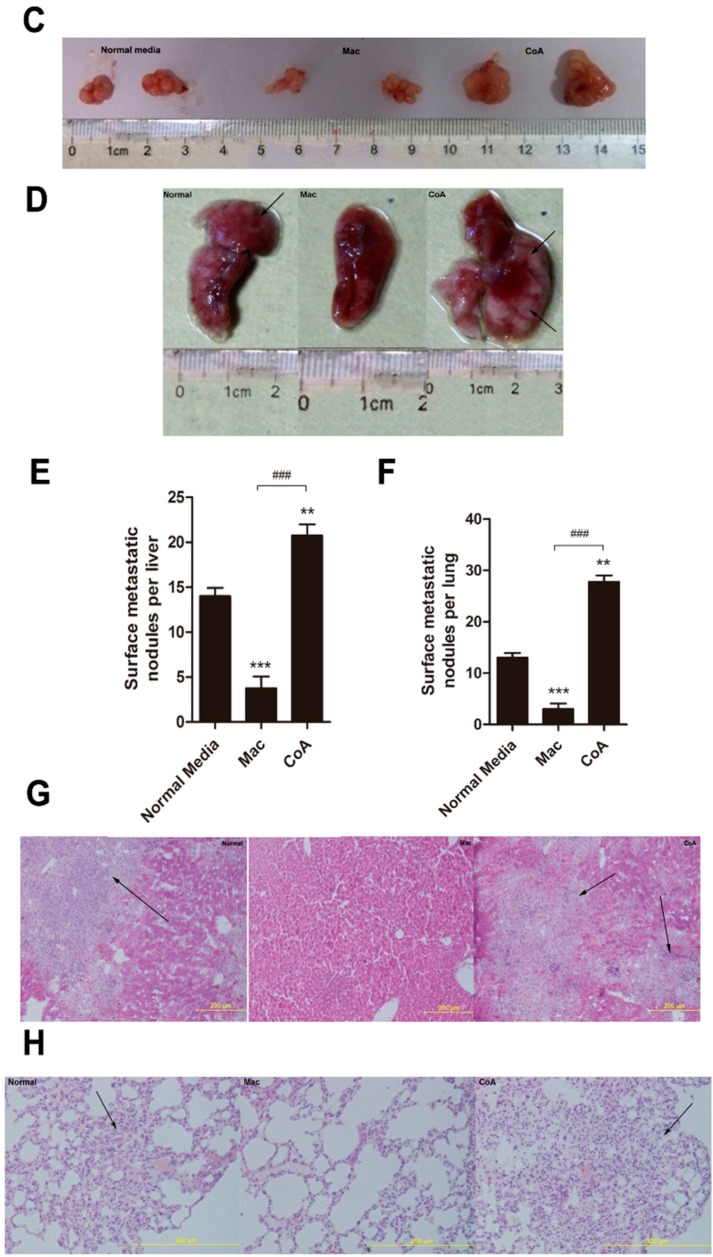
*In vivo* tumorigenicity and metastatic assay. (**A**) Growth curves for tumors generated by MCF-7 cells grown in three types of conditioned media. The width and diameter of each tumor were measured using calipers, and tumor volume was calculated using the formula ½ × a × b^2^, where “a” is the longer tumor axis and “b” is the shorter tumor axis; (**B**) Tumor weight was measured after excision from mice,*n* = 5; (**C**) Images of tumors from the three groups of mice; (**D**) Macroscopic view of nodules in the lungs from the three groups of mice; (**E**,**F**) Quantification of the metastatic nodules in the three groups of mice (*n* = 5); (**G**,**H**) Hematoxylin-eosin (HE) staining of paraffin sections from livers and the lungs of the three groups of mice. Metastases are indicated by the black arrows. ** *p* < 0.01, *** *p* < 0.001 *vs.* Normal media group; **^###^** *p* < 0.001 *vs.* Mac group.
